# Time-dependent association between omeprazole and esomeprazole and hospitalization due to hyponatremia

**DOI:** 10.1007/s00228-022-03423-x

**Published:** 2022-11-15

**Authors:** Issa Issa, Jakob Skov, Henrik Falhammar, Jan Calissendorff, Jonatan D. Lindh, Buster Mannheimer

**Affiliations:** 1 Department of Clinical Science and Education at Södersjukhuset, Karolinska Institutet, Södersjukhuset, SE-11883 Stockholm, Stockholm Sweden; 2 Department of Molecular Medicine and Surgery, Karolinska Institutet, Stockholm, Sweden; 3 Department of Medicine, Karlstad Central Hospital, Karlstad, Sweden; 4 Department of Endocrinology, Karolinska University Hospital, Stockholm, Sweden; 5 Department of Laboratory Medicine, Division of Clinical Pharmacology, Karolinska University Hospital Huddinge, Karolinska Institutet, Stockholm, Sweden

**Keywords:** Hyponatremia, Omeprazole, Esomeprazole, SIADH, PPI, Adverse effect

## Abstract

**Purpose:**

The aim of this study was to explore the time-course of hospitalization due to hyponatremia associated with omeprazole and esomeprazole.

**Methods:**

In this register-based case–control study, we compared patients hospitalized with a main diagnosis of hyponatremia (*n* = 11,213) to matched controls (*n* = 44,801). We used multiple regression to investigate time-related associations between omeprazole and esomeprazole and hospitalization because of hyponatremia.

**Results:**

The overall adjusted OR (aOR) between proton pump inhibitor (PPI) exposure, regardless of treatment duration and hospitalization with a main diagnosis of hyponatremia, was 1.23 (95% confidence interval CI 1.15–1.32). Exposure to PPIs was associated with a prompt increase in risk of hospitalization for hyponatremia from the first week (aOR 6.87; 95% *CI* 4.83–9.86). The risk then gradually declined, reaching an aOR of 1.64 (0.96–2.75) the fifth week. The aOR of ongoing PPI treatment was 1.10 (1.03–1.18).

**Conclusion:**

The present study shows a marked association between omeprazole and esomeprazole and hyponatremia related to recently initiated treatment. Consequently, newly initiated PPIs should be considered a potential culprit in any patient suffering from hyponatremia. However, if the patient has had this treatment for a longer time, the PPI should be considered a less likely cause.

## Introduction

Hyponatremia is a condition with consequences that range from mild headache and weakness to life-threating brain edema and coma [1]. The prevalence of hyponatremia differs depending on the studied population, but is estimated to be around 30% in hospitalized patients [1]. A wide range of causes can result in hyponatremia including diseases and organ dysfunctions such as pneumonia, liver cirrhosis, and heart failure [2]. However, hyponatremia can also be drug-induced [3]. The list of triggering substances is long and includes several commonly prescribed drugs such as thiazides [4], antiepileptic drugs [5], and antidepressants [6]. Proton pump inhibitors (PPIs) have also been associated with hyponatremia [7–11].

PPIs have been used as first-line treatment and prophylaxis of peptic ulcer and gastro-esophageal reflux disease (GERD) since the late 1980s [12]. Omeprazole and its S-isomer esomeprazole are the most widely used PPIs today and both compounds are generally well tolerated [13, 14]. Using a population-based approach, we have previously explored the association between hospitalization due to hyponatremia and the use of PPIs, showing that the risk of hospitalization was primarily increased in those that had initiated the treatment within the last 90 days [7, 8]. Detailed knowledge on the time course would be useful to aid clinicians in distinguishing a causal relationship from a spurious association. The aim of this study was to explore the time-course of hospitalization due to hyponatremia associated with omeprazole and esomeprazole.

## Methods

This retrospective case–control study investigated the association between omeprazole as well as esomeprazole exposure and hospitalization due to hyponatremia and was based on the entire adult population in Sweden. Omeprazole and esomeprazole are by far the two most commonly prescribed PPIs [12–14], collectively referred to as PPIs throughout this article. The index date was defined as the date of admission for each patient. Cases were defined as 18 years old or older persons hospitalized with a first-ever (since 1st of January 1997) main diagnosis of hyponatremia (E87.1) or syndrome of inappropriate antidiuretic hormone secretion (E22.2) between the 1st of October 2006 and the 31st of December 2014 as identified in ICD-10; the tenth revision of the International Classification of Diseases. Data on the ICD-10-encoded diagnoses were retrieved from the National Patient Register (NPR). For each hyponatremic case, four controls without prior diagnosis of hyponatremia were randomly identified in the Total Population Register. Cases and controls were matched for age, gender, and county of residence. The study covered information on diagnoses from the 1st of January 1997 to the 31st of December 2014. Information on drug exposure was retrieved from the Swedish Prescribed Drug Register (SPDR) that includes personal information on all prescription medications sold in Sweden since the 1st of July 2005 [14]. The Swedish longitudinal integration database for health insurance and labor market studies register (LISA) was used to collect information about the patients’ socio-economic status. The process has been described in more detail elsewhere [5, 6].

### Variables

Omeprazole and esomeprazole were identified by the anatomical therapeutic chemical (ATC) by codes A02BC01, A02BC05, A02BD06, and M01AE52. Exposure to these PPIs was defined as a drug prescription dispensed within 90 days before the index date. New PPI exposure was defined as a prescription of PPIs dispensed within 90 days prior to the index date, without any PPI dispensations during the preceding 12 months. Ongoing use was defined as filled prescriptions of PPI both within the last 90 days prior to the index date and the 12-month period preceding these 90 days. The duration of new PPI usage was further divided into weeks (1–13 weeks) based on date of the first PPI dispensation. Three registers (NPR, SPDR, and LISA) were used to gather data on potential confounders, which encompassed co-morbidities, medications used during the study period, and socioeconomic status. See Table 1 for a summary of all studied variables (both potential confounders and previous exposure).

**Table 1:** Variables used in the multiple regression analysis with their definitions

**Variables**	**Codes**
**Medications of main interest**	ATC codes starting with:
Omeprazole	A02BC01
Esomeprazole	A02BC05, A02BD06, M01AE52
**Other PPIs**	
Pantoprazole	A02BC02
Lansoprazole	A02BC03
Rabeprazole	A02BC04
**Anticonvulsants**	
Carbamazepine	N03AF01
Oxcarbazepine	N03AF02
Phenytoin	N03AB02
Valproate	N03AG01
Lamotrigine	N03AX09
Levetiracetam	N03AX14
Gabapentin	N03AX12
**Diuretics and medications acting on the renin–angiotensin pathway**	
Furosemide	C03C
Thiazides	C03A, C09BA, C09DA, C03EA
Agents acting on the renin–angiotensin system	C09
**Antibiotics**	
Fluoroquinolones	J01MA
Macrolides	J01FA
Trimethoprim sulfamethoxazole	J01EE
**Antidepressants**	
Serotonin reuptake inhibitors	N06AB
Tricyclic antidepressants	N06AA
Other antidepressants	N06AX
**Other medications**	
Amiodarone	C01BD01
Desmopression	H01BA02
Antipsychotics	N05A excluding N05AN
Lithium	N05AN
**Comorbidities**	ICD10 codes starting with:
Renal diseases	
Renal insufficiency	N17-19, procedure codes DR016, DR024, KAS00, KAS10, KAS20
Infections	
Sepsis	A41 ≤ 90 days from index date, R65
Pneumonia and empyema	J18 ≤ 90 days from index date, J86
Meningitis, encephalitis and cerebral abscess	G00–G07 ≤ 90 days from index date
Heart and vascular diseases	
Ischemic heart disease, recent	I20–24 ≤ 90 days from index date
Ischemic heart disease, old	I20–24 > 90 days from index date, I25
Congestive heart failure	I50
Cerebrovascular diseases, recent	I60–64 ≤ 90 days from index date
Cerebrovascular diseases, old	I60–64 > 90 days from index date, I69
Gastrointestinal diseases	
Pancreatic disease	K85, K860-1
Inflammatory bowel disease	K50–51
Liver diseases	K70–77 procedure codes JJB, JJC
**Other diseases**	
Hypothyroidism	E03, E06.3
Malnutrition	E43.9, E41.9
COPD	J44
Pulmonary embolism	I26
Malignancy	C
Alcoholism	ATC: N07BB03, N07BB04, N07BB01, N07BB05, N07BB ICD10: E244, F10, G312, G621, G721, I426, K292, K70, K860, O354, P043, Q860, T51, Y90–91, Z502, Z714
Polydipsia	R63.1
Adrenal insufficiency	ATC: H02AA, H01BA ICD10: E27.1, E27.2, E27.3, E27.4, E25
Diabetes mellitus	ATC: A10 ICD10: E10–E14
Socioeconomic factors/frailty	
Education	Increasing levels of education from 1 to 6, ordinal variable
Income	Annual income in SEK, continuous variable
Unemployment	Number of years, continuous variable
Drug usage	Number of dispensed drugs 90 days prior to index date, categorized into < 4, 4–7, 8–12, and > 12 drugs
Earlier periods of hospitalization	≥ 3 days

*ATC* anatomical therapeutic chemical, *COPD* chronic obstructive pulmonary disease, *ICD* international classification of diseases, *PPI* proton pump inhibitors

### Statistical analysis

The association between PPI and hospitalization due to hyponatremia was analyzed using logistic regression. Newly initiated (encoded as weeks of exposure as discussed above) and ongoing PPI exposure were included as independent variables, with and without adjustment for potential confounders. Associations were reported as unadjusted and adjusted odds ratios (ORs), with 95% confidence intervals (95% *CI*). *P*-values < 0.05 were considered statistically significant. For all analyses, R version 3.6.1 was used [15].

## Results

Throughout the study period, 11,213 patients with a main diagnosis of hyponatremia (cases) and 44,801 matched controls were identified. The mean age was 76 years and 72% were women (Table 2). Cases suffered from more comorbidities than controls — hypertension, diabetes mellitus, malignancy, and ischemic heart disease in particular. In addition, cases used more prescribed drugs than controls and more often had a history of hospitalization (Table 2).

**Table 2:** Medical characteristics (selection of variables from Table 1) and prescription of omeprazole or esomeprazole

	**Number (%) of total cases (*n* = 11,213)**	**Number (%) of total controls (*n* = 44,801)**
Age, years (median interquartile range)	76 (65;84)	76 (65;84)
Female gender	8074 (72.0)	32,254 (72.0)
**Diagnosis**		
Malignancy	3096 (27.6)	9149 (20.4)
Ischemic heart disease, recent	498 (4.4)	405 (0.9)
Ischemic heart disease, old	1918 (17.1)	6072 (13.6)
Diabetes mellitus	1939 (17.3)	5277 (11.7)
Alcoholism	1764 (15.7)	833 (1.9)
Congestive heart failure	1453 (13.0)	3533 (7.9)
Cerebrovascular disease, recent	218 (1.9)	164 (0.3)
Cerebrovascular disease, old	1299 (11.6)	3410 (7.6)
COPD	1125 (10.0)	1576 (3.5)
Hypothyroidism	1139 (10.2)	1994 (4.5)
Renal disease	489 (4.4)	888 (2.0)
Adrenal insufficiency	460 (4.1)	300 (0.7)
Liver disease	421 (3.8)	332 (0.7)
Pancreatic disease	252 (2.2)	395 (0.9)
IBD	221 (2.0)	444 (0.1)
**Medications**		
Antidepressants	2817 (25.1)	5745 (12.8)
Antipsychotics	772 (6.9)	1096 (2.4)
Antiepileptic drugs	1061 (9.5)	1128 (2.5)
Furosemide	1735 (15.5)	5487 (12.2)
Thiazide diuretics	4364 (38.9)	6103 (13.6)
Lipid-lowering drug	2314 (20.6)	7525 (16.7)
**Proxy for frailty**		
Number of dispensed drugs 90 days prior to index date		
< 4 drugs	2215 (19.8)	22,892 (51.1)
4–7 drugs	3421 (30.5)	12,967 (28.9)
8–12 drugs	3558 (31.7)	7010 (15.6)
> 12 drugs	2019 (18.0)	1.932 (4.3)
Number of patients with ≥ 1 hospitalization ≥ 3 days duration	4852 (43.2)	9477 (21.2)
Prescription of PPI		
Any PPI	3119 (27.8)	5774 (12.9)
Omeprazole	2628 (23.4)	4841 (10.8)
Esomeprazole	379 (3.4)	529 (1.2)
Omeprazole or esomeprazole	2924 (26)	5323 (11.9)
Other PPIs	238 (2.1)	477 (1.1)

Recent during the 90 days before index date; *IBD* inflammatory bowel disease, *PPI* proton pump inhibitors

**Fig. 1:**
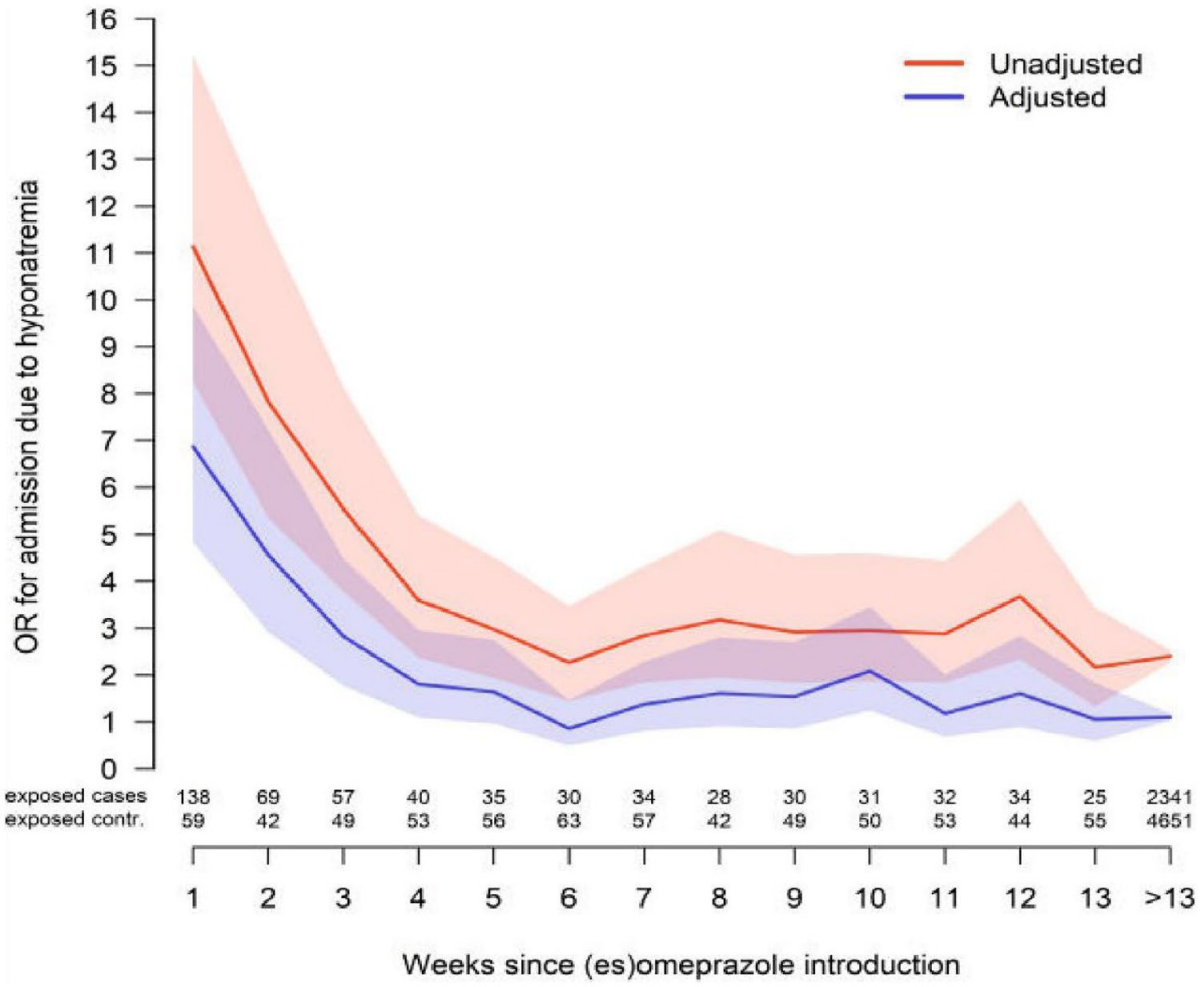
Odds ratios (OR) and adjusted odds ratios (aOR) for hospitalization secondary to hyponatremia on a weekly basis (95% confidence interval)

There was a significant association between PPIs exposure regardless of treatment duration and hospitalization with a main diagnosis of hyponatremia, with an adjusted *OR* (aOR) of 1.23 (95% *CI* 1.15–1.32). The time-dependent association between the start of PPI-treatment and the date of hospitalization is depicted in Fig. 1. Overall, crude *ORs* were higher than adjusted *ORs*. Exposure to PPIs was associated with a prompt increase in risk of hospitalization for hyponatremia from the first week (aOR 6.87; 95% *CI* 4.83–9.86); the risk then gradually declined reaching an aOR of 1.64 (0.96–2.75) by the fifth week. The aOR of the ongoing PPI treatment was 1.10 (1.03–1.18).

## Discussion

Using population-based retrospective data, we explored the time relation between initiation of omeprazole and esomeprazole and hospitalization secondary to hyponatremia in Sweden. The risk was substantially increased from the first week of using PPI and decreased reaching a near normal level by week 5. Ongoing PPI exposure was not associated with the same risk of hyponatremia requiring hospitalization (aOR 1.1).

Previous evidence on the association between PPIs and hyponatremia is scarce and mostly based on small studies [9–11]. Buon et al. investigated the prevalence of PPI in elderly individuals with and without hyponatremia (defined as moderate hyponatremia 123–134 mmol/L) [9]. The authors concluded that PPI use was more common among hyponatremic patients. However, due to the limited number of individuals studied (*n* = 145), the estimate was rather imprecise (*OR* 4.4 [95% *CI* (1.8–11]) [9]. Makunts et al. took advantage of post–marketing safety data for PPIs from the United States Food and Drug Administration (FDA) Adverse Event Reporting System records [10]. The association between omeprazole and esomeprazole and hyponatremia differed substantially (*OR* 7 vs *OR* 0.6) which is remarkable considering that omeprazole is a racemic mixture of esomeprazole and the S-isomer of omeprazole. However, with data being based on self-reporting and an analysis that did not control for possible confounders, the results should be interpreted with caution [10]. None of these studies explored the temporal aspects of PPIs and subsequent hyponatremia. The only study exploring the temporal aspects is our previous study, which was based on the same cohort as the present one and found that initiated PPIs (within 90 days) were increased (*OR* 2.8) while the ongoing use was only slightly increased compared to controls (*OR* 1.1) [7, 8]. In this study, no large difference was observed in aOR between omeprazole and esomeprazole: 1.67 (95% *CI* 2.37–3.01) for omeprazole and 2.89 (95% *CI* 2.21–3.79) for esomeprazole [7, 8]. We did not compare these two PPIs in our study.

Several drug-related side-effects do show a temporal relation with duration of treatment that may represent a vulnerability on behalf of the affected patient. Regarding hyponatremia, such a temporal relationship has been noted for thiazides, antiepileptic drugs, venlafaxine, and selective serotonin reuptake inhibitors (SSRIs), tramadol and codeine [4–6, 16, 17]. Thus, SSRIs were associated with dramatically elevated risk for hyponatremia from the first week (aOR 29) then gradually declining and normalizing after a few months [16]. Also thiazide-induced hyponatremia has a clear temporal relationship with a risk being substantially elevated immediately after initiation and then declining [4].

The mechanism by which PPI may cause hyponatremia is still unclear but has been attributed to the retention of fluid secondary to an inappropriate ADH secretion (syndrome of inappropriate antidiuretic hormone secretion SIADH) [1, 2]. Knowledge on the temporal association is interesting as it may help distinguish a spurious relationship from a causal one and potentially guide the clinician when meeting a patient with PPI treatment also suffering from hyponatremia. The present study therefore aimed to investigate the time course of PPI–associated hyponatremia in greater detail. This time, we focused on omeprazole and esomeprazole only, the two most commonly used PPIs, exploring the week-by-week association. The association was immediately substantially elevated reaching an adjusted *OR* of 7 and then decreased to near normal levels after a month of treatment.

“There were some limitations to our study. Due to the retrospective register-based approach we lacked information on medication adherence. Thus, although we did have information on the time of drugs being dispensed, we could not ascertain if or when they were consumed. Furthermore, apart from being dispensed from pharmacies, omeprazole and esomeprazole are also sold over the counter (OTC). As OTC drugs are not recorded by the registers used, we might have underestimated the proportion of individuals exposed for PPIs among both cases and controls which may have introduced bias. However, in contrast to prescribed drugs, OTC medications are not reimbursed in Sweden, so if more regular use is required it can be assumed to be prescribed. Moreover, we adjusted for the effects of a range of co-morbidities, medications and factors that mirror socioeconomic status, listed in table 1. However, due to the register-based observational approach, we cannot exclude some level of residual confounding. For example, while 15.7% of the cases were associated with previous drug use or diagnosis indicating alcoholism the corresponding proportion among controls was 1.9%. Although the multivariate analysis was adjusted for this imbalance, the ATC-codes and ICD-codes used to define excessive alcohol use might not have mirrored the true proportion which may have introduced bias. The results might therefore be interpreted with caution. Furthermore, the database used lacked information on sodium levels. However, a previous validation of the present cohort showed that the mean sodium concentration observed in admitted patients was 121 mmol/L, and that the majority (89%) of patients were judged to be admitted primarily due to low sodium, indicating a high relevance of the outcome used [5].”

The prompt temporal relationship between initiating PPI and hospitalization due to hyponatremia suggests a causal association. However, some of the association may be mediated through the condition for which the initiated PPI was used such as esophagitis or a peptic ulcer, in other words confounding by indication [12–14].

The present study has important clinical implications. Newly started PPI treatment should be suspected as the underlying cause in any patient presenting with hyponatremia. The suspicion is strengthened in the absence of concomitant severe disease, such as a bleeding ulcer. However, if a patient has had this treatment for a longer time, other potential causes should be thoroughly investigated before the PPI treatment is abandoned.

In conclusion, the study shows a marked association between omeprazole/esomeprazole and hyponatremia exclusively related to recently initiated treatment. Consequently, newly initiated PPIs should be considered the culprit in any patient suffering from hyponatremia. However, if patients had this treatment a longer time, the PPI should be considered a less likely cause.

## Data Availability

Data will be made available due to reasonable requests.
